# 

**Published:** 1851-03

**Authors:** 


### CONTENTS

OF THE

### MEDICAL EXAMINER.

NEW SERIES—NO. LXXV.—MARCH, 1851.

### ORIGINAL COMMUNICATIONS.

Lectures on Scarlet Fever. By Caspar Morris, M. D., late Lecturer on Practical Medicine in the Philadelphia Medical Institute, - 141
Case of Poisoning by Arsenious Acid. By G. W. Brown, M. D., of Port Carbon, Pa. - - - - - - - 153
Remarks on the use of Bromide of Iron. By Edward Gillespie, M. D., of Brady's Bend, Pa. - - - - - - - 158
Case of Wound of the Stomach. By Robert K. Smith, M. D., of Darby, Pa. - - - - - - - 162

### CLINICAL REPORTS.

Clinic of the Jefferson Medical College. Service of Profs. Mütter and Pancoast, - - - - - - - 164
Summary of the Course on Demonstrative Surgery, in the University of Pennsylvania, from Jan. 25th to Feb. 22d., - - - 174

Cases from the dispensary practice of Pennsylvania College. Reported by Mr. H. J. Richards, Clerk of the Medical and Surgical dispensary, - - - - - - 182

### BIBLIOGRAPHICAL NOTICES.

A Practical Treatise on Dental Medicine, being a compendium of Medical Science, as connected with the study of Dental Surgery. By Thomas E. Bond, A. M. M. D., Professor of Special Pathology and Therapeutics in the Baltimore College of Dental Surgery, - - - - - - - 186
First Principles of Medicine. By Archibald Billing, M. D., A. M., F. R. S., formerly Professor of Clinical Medicine and Physician of the London Hospital, - - - - - 189
Review of Chemistry for Students. Adapted to the courses as taught in the Principal Medical Schools in the United States. By John G. Murphy, M. D., Philadelphia, - - 190

### EDITORIAL.

Rank of Medical Officers in the Navy, - - - - 191
Medical Certificates for Life Insurance offices, - - - 198

### MEDICAL NEWS.

Case of Fistula of the Stomach, - - - - - 199
Deaths of Distinguished Physicians, - - - - - 199
Delegates to the American Medical Association, - - - 199
Catalogues of Students, - - - - - - 200
Proportion of Physicians to the Population of Virginia, - - 200
Professor of Chemistry in Harvard University, - - - 200
Adulterated Drugs in New York, - - - - - 200
Assistant Surgeons United States Navy, - - - - 200

### RECORD OF MEDICAL SCIENCE.

### PATHOLOGY AND PRACTICE OF MEDICINE.

The Alum Treatment of Lead Colic, - - - - 201
Cases of the Termination of Acute Rheumatism in Suppuration, - 201

Acute Articular Rheumatism treated with Lemon-Juice, - - 202
Expulsion of the Tape-worm by the Oil of the Male Fern, - - 203

SURGERY.

Case of Dislocation of the Scaphoid Bone of the Foot, - - 203
Case of Luxation of the Penis, - - - - - 204
Repeated Injections of Iodine in the Treatment of Encysted Tumors, 205
Collodion applied to Burns, - - - - - - - 205
Extirpation of Goître, - - - - - - 205

OBSTETRICS.

On Lactation, - - - - - - - - 206

